# Visual continuous recognition reveals behavioral and neural differences for short- and long-term scene memory

**DOI:** 10.3389/fnbeh.2022.958609

**Published:** 2022-09-15

**Authors:** Timothy M. Ellmore, Chelsea Reichert Plaska, Kenneth Ng, Ning Mei

**Affiliations:** ^1^Department of Psychology, The City College of the City University of New York, New York, NY, United States; ^2^Behavioral and Cognitive Neuroscience, The Graduate Center of the City University of New York, New York, NY, United States

**Keywords:** EEG, working memory, naturalistic stimuli, ERP, signal detection, retention intervals

## Abstract

Humans have a remarkably high capacity and long duration memory for complex scenes. Previous research documents the neural substrates that allow for efficient categorization of scenes from other complex stimuli like objects and faces, but the spatiotemporal neural dynamics underlying scene memory at timescales relevant to working and longer-term memory are less well understood. In the present study, we used high density EEG during a visual continuous recognition task in which new, old, and scrambled scenes consisting of color outdoor photographs were presented at an average rate 0.26 Hz. Old scenes were single repeated presentations occurring within either a short-term (< 20 s) or longer-term intervals of between 30 s and 3 min or 4 and 10 min. Overall recognition was far above chance, with better performance at shorter- than longer-term intervals. Sensor-level ANOVA and *post hoc* pairwise comparisons of event related potentials (ERPs) revealed three main findings: (1) occipital and parietal amplitudes distinguishing new and old from scrambled scenes; (2) frontal amplitudes distinguishing old from new scenes with a central positivity highest for hits compared to misses, false alarms and correct rejections; and (3) frontal and parietal changes from ∼300 to ∼600 ms distinguishing among old scenes previously encountered at short- and longer-term retention intervals. These findings reveal how distributed spatiotemporal neural changes evolve to support short- and longer-term recognition of complex scenes.

## Introduction

Humans have a remarkable capacity for remembering complex visual information. Early behavioral studies demonstrated that adults and children can recognize large sets of visual stimuli after a single exposure (Shepard, 1967; Standing et al., 1970; Brown and Scott, 1971). While speed of recognition for pictures tends to be slower than for verbal material, reaction times for a range of learning set sizes indicate fast memory search (Standing, 1973). Picture recognition is also highly flexible, with subjects able to discriminate in forced choice paradigms between targets and distractors using perceptual and ecphoric similarity (Tulving, 1981). Early studies of visual memory capacity often mixed objects with travel slides containing complex naturalistic visual scenes. Subsequent research compared encoding of complex scenes with edited versions of scenes that contained a common feature (e.g., a door) and found memory performance for non-edited original photographs was close to 85%. When scene details were removed, performance dropped by as much as 20%, suggesting that visual details in scenes contribute positively to long-term memory (Vogt and Magnussen, 2007).

Other findings support that subjects successfully maintain detailed representation of thousands of images (Brady et al., 2008). When the number of exemplars from different categories is controlled for during the study of large picture sets, the capacity to remember visual information in long-term memory varies more with conceptual structure than perceptual distinctiveness. Images from object categories with conceptually distinctive exemplars show less interference as the number of exemplars is increased (Konkle et al., 2010). High capacity picture memory would appear to be at odds with the traditional view that working memory capacity is limited to three or four items. The ability to recognize complex images after short retention intervals would seem to require a larger capacity temporary store, especially if complex details are used. When maintenance using a rehearsal strategy is prevented by using rapid serial visual presentation, memory capacities of up to 30 retained pictures for 100 item lists are obtained, which suggests humans have a larger capacity temporary memory store when proactive interference is minimized (Endress and Potter, 2014).

Scalp EEG has been used to demonstrate fast, parallel processing of complex scenes. In a go/no-go task in which subjects must determine whether a briefly presented scene contains an animal or not, a frontal event related negativity develops on no-go trials approximately 150 ms after stimulus onset (Thorpe et al., 1996). Event related potentials (ERPs) reflect the visual category of a scene as early as 75–80 ms post-stimulus, but are not correlated with behavior until around 150 ms (Vanrullen and Thorpe, 2001). Subjects are as fast at responding to two simultaneously presented scenes as to a single one (Rousselet et al., 2002) demonstrating parallel processing, but behavior and ERPs suffer a processing cost when up to four scenes are presented simultaneously (Rousselet et al., 2004). For biologically relevant scenes, fronto-central ERPs begin to diverge from other stimulus categories around 185 ms after stimulus onset, with a later divergence in parietal regions (Anokhin et al., 2006). Scene recognition is state-dependent and can be modulated by alcohol intoxication (De Cesarei et al., 2006), which reduces early differential ERP activity occurring 150–220 ms when discriminating targets from non-target distractors. An early marker of scene-specific processing was found in a recent study which reported that the first ERP component to evoke a stronger response to real-world scenes compared to other categories is the P2, peaking approximately 220 ms after stimulus onset (Harel et al., 2016).

Intracranial EEG and fMRI studies identify spatiotemporal aspects of scene processing. An intra-cerebral study found early posterior parahippocampal gyrus gamma (50–150 Hz) activity between 200 and 500 ms when subjects passively viewed scenes (Bastin et al., 2013). Functional MRI activity in both lateral occipital area (LOC) and parahippocampal place area (PPA) can be harnessed to classify scenes accurately. PPA activity confuses scenes that have similar spatial boundaries, while LOC activity confuses scenes that have similar content (Park et al., 2011). Recent work extends the role for occipital place area by demonstrating it can predict pathways for movement in novel scenes (Bonner and Epstein, 2017). It has also been demonstrated recently that humans do not segment a scene into objects but instead use global, ecological properties like navigability and mean depth (Greene and Oliva, 2009). Neural evidence also shows that contrast energy and spatial coherence modulate single-trial ERP amplitudes early (100–150 ms), with spatial coherence influencing later activity up to 250 ms (Groen et al., 2013).

While previous behavioral studies demonstrate that scene memory is high capacity and long-lasting and previous neural studies have characterized scene-specific neural changes, a gap in knowledge remains: what are the spatiotemporal neural dynamics that distinguish short-term (∼20 s) from longer-term scene memory? In the present study, we asked three main questions: When do neural evoked response potential (ERP) scalp topography amplitude changes distinguish scenes from scrambled perceptual input? How do neural changes differentiate new from previously presented old scenes? How do neural changes differ for old scenes previously presented at a short-term retention interval resembling working memory, and what are the changes for longer-term intervals extending to minutes, beyond the temporal limits of working memory?

## Materials and methods

### Subjects

Subjects were healthy adults between the ages of 18 and 29 with normal or corrected-to-normal vision and the ability to make button presses. Participants were excluded if they did not speak English. Each participant provided written informed consent and completed study procedures according to a protocol approved by the Institutional Review Board of the Human Research Protection Program. Participants were compensated $15 per hour for participation. All participants completed the scene memory task during high density scalp electroencephalography (HD-EEG). This study included a total of 27 subjects (mean age 21.33, std. age 2.92, range 18–29, 9 males, 1 left-handed).

### Experimental design

Subjects completed four 20-min runs of a visual continuous recognition task (VCRT) during a single session with HD-EEG recording. The VCRT stimuli consisted of color scenes and phase-scrambled scenes (Supplementary Figure 1). Scenes were 24-bit color images randomly sampled from the SUN database (Xiao et al., 2010). Only a small fraction (618) of all the SUN database pictures were used in the present study. Care was taken to sample pictures of outdoor scenes with no clearly visible faces. The task was programmed in Visual C + + with graphic presentation optimized by pre-loading as texture maps all stimuli into video RAM using OpenGL. Each stimulus was presented for 1,400 ms with jittered interstimulus intervals (ISI). A total of 1,228 stimuli were shown during the 80-min EEG testing session (∼15.35 stimuli per minute). Stimuli consisted of 305 scrambled scenes, 618 new scenes, with 309 of the new scenes sub-divided among three old conditions and subsequently repeated one more time (1) within 20 s (Old_1_), (2) within 30 s and 3 min (Old_2_), and (3) between 4 and 10 min (Old_3_). The stimulus design matrix for each VCRT run is shown in Supplementary Figure 2.

Each scene was displayed on a 27-inch LED monitor with a refresh rate of 60 hertz (Hz) and a screen resolution of 1920-by-1080. Participants sat 83.5 cm from the monitor and maintained stable viewing using a combined forehead/chin rest. Each scene measured 800-by-600 pixels on the screen, and from the subject’s point of view occupied a horizontal viewing angle of 17.2 degrees and a vertical viewing angle of 12.7 degrees. The EEG recordings took place within a sound-attenuated booth (IAC acoustics) to minimize auditory and visual distractions. Subjects made one of two button (green = old; red = new) responses with their thumb using a fiber optic response device (fORP 904, Current Designs, Inc.) held in their right hand.

### Behavioral analysis

Analyses of VCRT behavioral data included computing subject accuracy in the form of percent correct in distinguishing old and new scenes. Signal detection analyses were also performed to assess each subject’s recognition sensitivity as follows. A hit was counted when an old scene was correctly classified as an old scene. A false alarm was counted when a new scene was incorrectly classified as an old scene. For each subject, total hits and false alarms were expressed as proportions and used to compute a measure of sensitivity as the difference in standardized normal deviates of hits minus false alarms: d-prime (d’) = *Z(hit rate) – Z(false alarm rate)*. The d-prime sensitivity measure represents the separation between the means of the signal and noise distributions, compared against the standard deviation of the signal or noise distributions (Stanislaw and Todorov, 1999).

Overall percent correct and d-prime were based on the ability to recognize scenes as old or new across the four 20-min blocks. Separate accuracy and d-prime measures were computed for each condition of old: Old_1_ (repeated presentation within < 20 s), Old_2_ (repeated presentation between 30 s and 3 min), and Old_3_ (repeated presentation between 4 and 10 min). Average subject response times were also computed for new, old, hits, misses, false alarms, and correct rejections. Repeated measures ANOVAs of accuracy, d-prime, and response times were performed using JASP 0.8.3.1 with *post hoc* tests and Bonferroni multiple comparisons corrections^1^.

### EEG acquisition

EEG data were sampled at 1 kHz using Pycorder software from 62 scalp locations using an active electrode system with an actiCHamp amplifier (Brain Products). Electrodes were placed at standard locations specified by an extended 10–20 system. The recording ground (Fpz) was located at the frontal midline and the recording reference was located at the left mastoid (TP9) leaving 61 scalp recordings. Two additional channels were designated for left (LOC) and right (ROC) vertical electrooculography (VEOG) recordings for subsequent isolation of eye blink artifacts.

Recordings to disk began after electrode impedances fell below 25 K Ohms. Although the standard convention is to reduce impedance to 5 K Ohms or below (Teplan, 2002), the actiCHamp system uses active electrodes with noise reducing techniques built into the amplifier to ensure that impedances under 25 K ohms are sufficient for interpretable signals. Channels with impedance values above 25 K ohms were interpolated using data from neighboring electrodes with impedances below 25 K ohms. An auxiliary channel was used to record from a photosensor placed directly on a corner of the LED monitor. A 10-by-10 pixel square located under the photosensor was programmed to change from white to black during the onset of each visual stimulus; it changed from black to white during stimulus offset. Recording changes in screen luminance from the photosensor at 1 kHz allowed for precise timing of stimulus onset and offset with respect to the recorded EEG data.

### EEG analysis

EEG signals for each participant were sampled in four separate “runs.” Each run lasted approximately 20 min and contained separate randomization schedules for the different task conditions. Short breaks (approximately 5 min) were taken between runs to mitigate any participant fatigue. The four runs for each participant were pre-processed separately using BESA Research v6.1. The steps in the processing of each run included:

1. Each channel’s signal was visually inspected to find, mark, and exclude the duration of all muscle artifacts.
2. A characteristic eye-blink was marked by finding an alternating deflection greater than 100 microvolts (μV) between the LOC and ROC signals.
3. A template matching algorithm was then used to find all eye blink artifacts on all channels and remove the component of variance accounted for by the eye blinks (Picton et al., 2000; Ille et al., 2002).
4. An automated artifact scan was conducted to isolate and exclude additional artifacts using amplitude (120 μV), gradient (75 μV), and low-signal (max. minus min) criteria (0.01). Participant’s data were used in further processing only if a minimum of 60% of trials in each run survived the final artifact scan.
5. The signal on each channel was high pass filtered (low cut 0.1 Hz, type forward, slope 6 dB/oct).
6. The EEG signals were re-referenced to a common average reference.
7. All four processed EEG runs for each participant were combined to produce one average evoked response potential (ERP) for each condition. During this final step, each signal was low pass filtered (high cut 40 Hz, type zero phase, slope 12 dB/oct) before export as an ASCII vectorized file. Each.avr file contained the average ERP for each condition, which was then used as input to a group ERP analysis in BESA Statistics 2.1.

Care was taken in the level and order of application of the low and high cutoff filters. Artifacts due to high-pass filtering can lead to systematic biases between conditions when a cut-off above the recommended maximum of 0.1 is used (Acunzo et al., 2012; Tanner et al., 2015). We applied low pass filtering as the last step so as not to distort average ERPs (Luck, 2014).

Following filtering and cleaning of EEG data, average evoked response potentials (ERPs) were computed for each condition (e.g., new, old (all), old_1_, old_2_, old_3_, and scrambled). The average ERPs for each condition were then used as input to group ERP statistical analyses performed with BESA Statistics v2.1 with appropriate multiple corrections across space and time (Maris and Oostenveld, 2007; Maris, 2012). Using this approach, statistical significance is assessed using non-parametric cluster permutation tests (*N* = 1,000). Group ANOVAs were followed by *post hoc* pairwise comparison tests of different conditions in which contiguous clusters in space and time of coherent F exceeding an *a priori* corrected *p*-value of less than or equal to 0.05 were deemed significant. Summed *F* values of the clusters are compared to a null distribution of F sums of random clusters obtained by permuting the data order across subjects. This controls for type I errors due to multiple comparisons. The null hypothesis of the permutation test assumes that the assignment of the conditions per subject is random and exchangeable. The idea behind data clustering used in combination with permutation testing is that if a statistical effect is found over an extended time period in neighboring channels, it is unlikely that the effect occurred by chance. For paired comparisons, a statistical effect can have a positive or negative direction and therefore positive and negative cluster values may be obtained. The positive or negative cluster value is the test statistic reported for each cluster, and the *p*-value reported is the one associated with that cluster based on permutation testing. For each of the 1,000 permutations, new clusters are determined and the corresponding cluster values are derived for each cluster. Based on the new distribution, the alpha error of the initial cluster value can be directly determined. For example, if only 2% of all cluster values are larger than the initial cluster value, the initial cluster has a 2% chance that the null hypothesis was falsely rejected. This cluster would then be associated with a *p*-value of 0.02. The time with respect to stimulus onset and the sensor locations of each cluster are reported in addition to the cluster value and *p*-value.

## Results

### Behavioral

**FIGURE 1:**
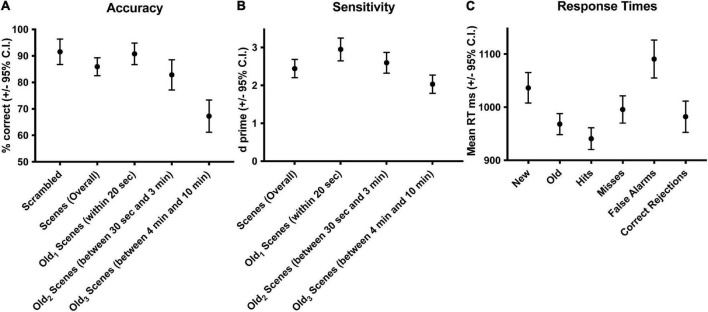
Behavioral accuracy and response times during scene recognition. Overall percent correct for new/old recognition was above chance (50%), was highest for the short-term interval and declined at the two longer term intervals **(A)**. A similar pattern was found for sensitivity **(B)**. Response times were longer for new compared to old scenes, fastest for hits and correct rejections, slower for misses, and slowest for false alarms **(C)**. Old_1_ is short-term interval up presented up to 20 s ago. Old_2_ is the longer-term interval between 30 s and 3 min. Old_3_ is the longer-term interval between 4 and 10 min.

Subjects performed well above chance (50%) discriminating old from new stimuli (85.7% correct, S.D. 8.5, Figure 1A). When old scene recognition was analyzed as a function of the three retention intervals, accuracy was best for the Old_1_ short-term interval and declined at each of the Old_2_ and Old_3_ longer-term intervals (repeated measures ANOVA, *F*_(2_,_56)_ = 186.3, *p* < 0.001, Figure 1A). A similar pattern was obtained when the dependent measure was sensitivity (d-prime) instead of percent correct (repeated measures ANOVA, *F*_(2_,_54)_ = 165.3, *p* < 0.001, Figure 1B). Subjects responded faster to old scenes compared to new scenes (old = 967.6 ms, s.d. 58.95 vs. new = 1012.1 ms, s.d. 79.45, paired *t*_(28)_ = 4.067, *p* < 0.001, Figure 1C). A signal detection breakdown of response times confirmed differences among hits (957.5 ms, s.d. 57.62), correct rejections (986.9 ms, s.d. 81.80), misses (1040.5 ms, 92.59 s.d.) and false alarms (1352.1 ms, 173.73 s.d.). A repeated measures ANOVA to compare the effect of type of response on the dependent variable response time was significant [*F*_(3_,_84)_ = 86.63, *p* < 0.001, Figure 1C]. *Post hoc* tests revealed that hits were significantly faster compared to both false alarms (*post hoc t* = −10.946, p_*bonf*_ < 0.001) and misses (*post hoc t* = −5.845, p_*bonf*_ < 0.001) but not correct rejections (*post hoc t* = −2.419, p_*bonf*=_0.134). Table 1 summarizes the behavioral results as a function of retention intervals and response times.

**TABLE 1:** Statistical summary of behavioral ERP analyses.

Behavioral analyses		

ANOVA	F (ndf, ddf)	*P*-value
Old_1_ vs. Old_2_. vs. Old_3_ (accuracy)	186.3 (2,56)	*p* < 0.001
Old_1_ vs. Old_2_. vs. Old_3_ (d-prime)	165.3 (2,54)	*p* < 0.001
Old_1_ vs. Old_2_. vs. Old_3_ (response time)	86.63 (3,84)	*p* < 0.001

***Post hoc* t**	***t***	**pbonf**

Hits vs. false alarms (RT, ms)	−10.946	*p* < 0.001
Hits vs. misses (RT, ms)	−5.845	*p* < 0.001
Hits vs. correct rejections (RT, ms)	−2.419	*p* = 0.134

The statistics and associated *p*-values are reported for each of the behavioral analyses. Old_1_ is short-term interval up presented up to 20 s ago. Old_2_ is the longer-term interval between 30 s and 3 min. Old_3_ is the longer-term interval between 4 and 10 min.

### Evoked response potentials ANOVAs

#### Event related potentials positivities distinguish new and old scenes from scrambled scenes

**FIGURE 2:**
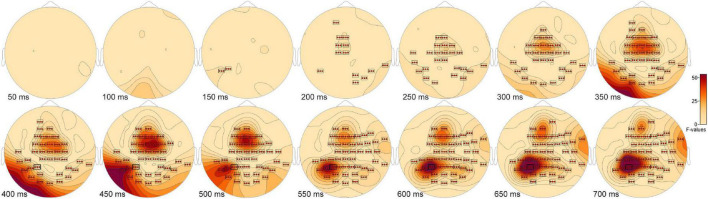
ANOVA F-map for the New vs. Old vs. Scrambled comparison. The F-map for the new vs. old. vs. scrambled comparison revealed a single spatially extended cluster of significant differences. Sensors with significant F are depicted as boxes with three red stars. Significant *F* values occurred as early as 138 ms after stimulus onset at left parietal sensor P7 and reached a maximum F at 684 ms at sensor P3.

The ANOVA F-map for the New vs. Old. vs. Scrambled comparison revealed a single spatially extended cluster of significant differences (Table 2, New vs. Old. vs. Scrambled, Cluster 1) beginning as early as 138 ms after stimulus onset at left parietal sensor P7 and reaching a maximum F of 90.92, *p* < 0.00001 at 684 ms at sensor P3 (Figure 2, red stars).

**TABLE 2:** Statistical summary of group ERP ANOVA permutation test results.

Permutation test results: ANOVA											

*New vs. Old vs. Scrambled*	Cluster value	*P*-value	Start (ms)	End (ms)	Max *F*-Value	Latency at Max (ms)	Channel at Max	Mean New	Mean Old	Mean Scrambled	
Cluster 1	743,209	*p* = 0.00000	138	1399	90.82	684	P3	0.119	0.143	0.121	

***Signal Detection: Hits vs. Misses vs. False Alarms vs. Correct Rejections***											

	**Cluster value**	***P*-value**	**Start (ms)**	**End (ms)**	**Max *F*-Value**	**Latency at Max (ms)**	**Channel at Max**	**Mean Hits**	**Mean Misses**	**Mean False Alarms**	**Mean Cor. Rej.**

Cluster 1	57,670	*p* = 0.001	309	900	27.78	599	C2	0.125	0.032	0.095	0.057
Cluster 2	1,726	*p* = 0.045	99	230	7.29	157	P2	1.947	2.068	1.400	2.091

*** Short- vs. Longer Retention Intervals: Old_1_ vs. Old_2_ vs. Old_3_***											

	**Cluster value**	***P*-value**	**Start (ms)**	**End (ms)**	**Max *F*-Value**	**Latency at Max (ms)**	**Channel at Max**	**Mean Old_1_**	**Mean Old_2_**	**Mean Old_3_**	

Cluster 1	224,754	*p* = 0.00000	228	1,399	20.019	317	FC1	−1.819	−2.845	−2.800	
Cluster 2	4,183	*p* = 0.059	469	747	11.255	645	TP10	−3.685	−2.693	−2.220	

The statistics and associated *p*-values are reported for each of the group ERP analyses. Sensor locations are named according to the extended international 10/20 system. Means for ERP conditions are in units of microvolts and are integrated across the entire temporal window indicated by start and end time. Old_1_ is short-term interval up presented up to 20 s ago. Old_2_ is the longer-term interval between 30 s and 3 min. Old_3_ is the longer-term interval between 4 and 10 min.

**FIGURE 3:**
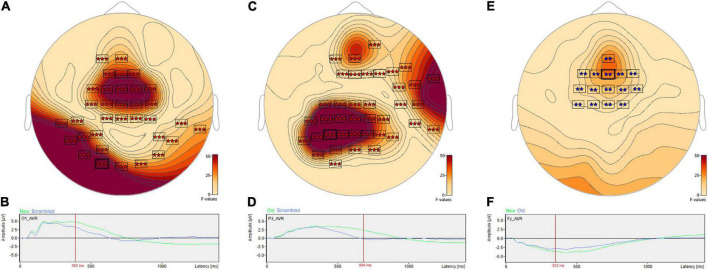
ANOVA *post hoc* pairwise comparisons for Old vs. Scrambled, New vs. Scrambled and New vs. Old. *Post hoc* pairwise comparisons revealed a single cluster of greater ERP positive amplitude for New compared to Scrambled at sensor O1 **(A)** at 392 ms after stimulus onset **(B)**. The Old-Scrambled comparison found a single cluster of greater positive amplitude for Old compared to Scrambled at sensor P3 **(C)** at 684 ms after stimulus onset **(D)**. The New-Old comparisons revealed two clusters of differences in amplitudes. The most significant cluster exhibited a less negative amplitude for Old compared to New at sensor Fz **(E)** at 352 ms after stimulus onset **(F)**.

*Post hoc* pairwise comparisons revealed a single cluster (Table 3, New vs. Scrambled) of significantly greater ERP positive amplitudes for New compared to Scrambled at sensor O1 (Figure 3A) at 392 ms after stimulus onset (Figure 3B). *Post hoc* pairwise comparisons revealed a single cluster (Table 3, Old vs. Scrambled) of significantly greater ERP positive amplitudes for Old compared to Scrambled at sensor P3 (Figure 3C) at 684 ms after stimulus onset (Figure 3D). *Post hoc* pairwise comparisons revealed two clusters of significant ERP amplitude differences between New and Old. The most significant cluster with the earliest difference (Table 3, New vs. Old, Cluster 1, *p* = 0.001) exhibited a less negative amplitude for Old compared to New at sensor Fz (Figure 3E) at 352 ms after stimulus onset (Figure 3F).

**TABLE 3:** Statistical summary of group ERP selected pairwise comparisons permutation test results.

Permutation test results: Selected pairwise comparisons											

*New vs. Old vs.* *Scrambled*	Cluster value	*P*-value	Start (ms)	End (ms)	Max *F*-Value	Latency at Max (ms)	Channel at Max	Mean New	Mean Old	Mean Scrambled	
*New vs. Old*											
Cluster 2	90,443	*p* = 0.001	177	876	32.08	352	Fz	−1.717	−0.982	NA	
Cluster 3	38,812	*p* = 0.001	194	856	23.03	651	TP10	2.873	2.069	NA	
*New vs. Scrambled*											
Cluster 1	1,082,230	*p* = 0.00000	139	1,399	78.13	392	O1	0.074	NA	0.062	
*Old vs. Scrambled*											
Cluster 1	779,757	*p* = 0.00000	270	1,399	160.28	684	P3	NA	0.141	0.104	

***Signal Detection: Hits vs. Misses vs. False Alarms vs. Correct Rejections***											

	**Cluster value**	***P*-value**	**Start (ms)**	**End (ms)**	**Max *F*-Value**	**Latency at Max (ms)**	**Channel at Max**	**Mean Hits**	**Mean Misses**	**Mean False Alarms**	**Mean Cor. Rej.**

*Hits vs. Misses*											
Cluster 2	55,996	*p* = 0.001	353	749	81.33	600	C2	0.346	−0.398	NA	NA
Cluster 3	8,335	*p* = 0.004	568	748	33.98	642	TP10	−1.352	−0.385	NA	NA
Cluster 4	4,654	*p* = 0.006	486	669	25.47	541	FT10	−1.784	−0.460	NA	NA
*Hits vs. False Alarms*											
Cluster 2	53,681	*p* = 0.001	324	760	66.18	549	C2	0.060	NA	−0.689	NA
Cluster 3	15,226	*p* = 0.002	450	736	35.35	650	TP10	−0.143	NA	0.833	NA
Cluster 4	10,289	*p* = 0.004	314	426	38.10	363	P8	2.161	NA	3.030	NA
*Hits vs. Cor. Rej.*											
Cluster 2	37,555	*p* = 0.001	323	679	44.67	549	C2	−0.090	NA	NA	−0.811
Cluster 3	9,725	*p* = 0.001	455	742	33.35	541	FT10	−1.418	NA	NA	−0.257
Cluster 4	2,666	*p* = 0.003	321	428	16.18	365	P8	2.682	NA	NA	3.430
Cluster 5	2,608	*p* = 0.003	681	745	14.93	710	CPz	0.944	NA	NA	0.435
Cluster 6	558	*p* = 0.005	376	409	12.91	393	PO7	2.462	NA	NA	3.135
Cluster 7	367	*p* = 0.008	548	585	11.04	562	FT9	−1.354	NA	NA	−0.165

***Short- vs. Longer Retention Intervals: Old_1_ vs. Old_2_ vs. Old_3_***											

	**Cluster value**	***P*-value**	**Start (ms)**	**End (ms)**	**Max *F*-Value**	**Latency at Max (ms)**	**Channel at Max**	**Mean Old_1_**	**Mean Old_2_**	**Mean Old_3_**	

*Old_1_ vs. Old_2_*											
Cluster 2	25,822	*p* = 0.001	230	544	31.92	311	FC1	−1.713	−2.462	NA	
Cluster 4	16,416	*p* = 0.001	228	517	18.91	393	P7	3.701	4.561	NA	
Cluster 5	4,920	*p* = 0.007	532	688	11.18	607	P2	1.993	1.444	NA	
*Old_1_ vs. Old_3_*											
Cluster 2	75,466	*p* = 0.001	229	762	34.76	333	FC1	0.255	NA	−0.466	
Cluster 3	8,025	*p* = 0.007	464	674	18.27	601	T7	−0.954	NA	0.258	
Cluster 5	3,907	*p* = 0.016	474	738	20.59	646	TP10	−1.933	NA	−0.682	
Cluster 6	3,590	*p* = 0.02	502	747	20.52	656	FT10	−5.520	NA	−3.684	
Cluster 7	3,343	*p* = 0.024	283	416	16.93	326	P6	2.464	NA	3.205	
*Old_2_ vs. Old_3_*											
Cluster 2	14,781	*p* = 0.001	635	975	22.49	667	P5	NA	1.399	0.797	
Cluster 3	6,808	*p* = 0.003	432	633	12.79	571	P5	NA	2.707	2.129	

The statistics and associated *p*-values are reported for each of the group ERP analyses. Sensor locations are named according to the extended international 10/20 system. Means for ERP conditions are in units of microvolts and are integrated across the entire temporal window indicated by start and end time. Old_1_ is short-term interval up presented up to 20 s ago. Old_2_ is the longer-term interval between 30 s and 3 min. Old_3_ is the longer-term interval between 4 and 10 min. This table lists selected significant post hoc pairwise comparisons occurring within a time window of 0 to 700 ms after stimulus onset. The complete set of significant post hoc pairwise comparisons occurring within the entire time window of 0 to 1,400 ms is included in the Supplementary Table.

#### Signal detection event related potentials analysis reveals a central positivity for hits

**FIGURE 4:**
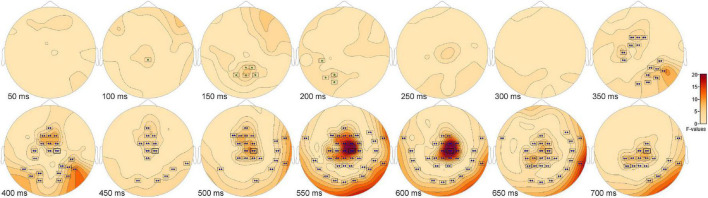
ANOVA F-map for the signal detection comparison. The F-map for the signal detection comparison revealed two spatially extended clusters of significant differences. The most significant cluster (*p* = 0.001, boxes with two blue stars) exhibited differences starting at 309 ms after stimulus onset and reached a maximum F of 27.78 at 599 ms at sensor C2. The other significant cluster (*p* = 0.045, boxes with one green star) showed differences earlier, starting at 99 ms after stimulus onset and reached a maximum F of 7.29 at 157 ms at sensor P2.

The ANOVA F-map for the signal detection comparison revealed two spatially extended clusters of significant ERP differences (Table 2, Hits vs. Misses vs. False Alarms vs. Correct Rejections). The most significant cluster (*p* = 0.001, blue stars, Figure 4) exhibited differences starting at 309 ms after stimulus onset and reached a maximum F of 27.78 at 599 ms at sensor C2. The other cluster (*p* = 0.045, green stars, Figure 4) exhibited differences earlier, starting at 99 ms after stimulus onset and reached a maximum F of 7.29 at 157 ms at sensor P2.

**FIGURE 5:**
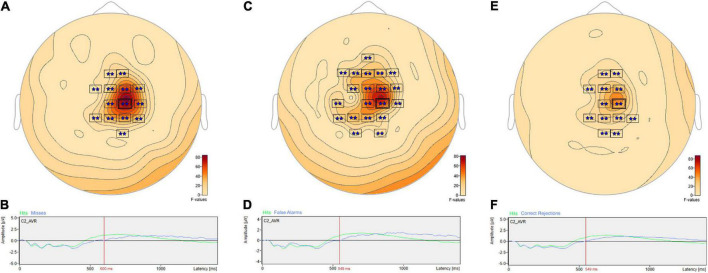
ANOVA *post hoc* pairwise comparisons for Hits vs. Misses, Hits vs. False Alarms and Hits vs. Correct Rejections. *Post hoc* pairwise comparisons revealed multiple clusters of significant differences between Hits vs. Misses, Hits vs. False Alarms and Hits vs. Correct Rejections. For Hits vs. Misses, significant differences were centered at sensor C2 **(A)** beginning at 353 ms and reaching a maximum *F* value of 81.33 at 600 ms with mean amplitudes being greater for hits than misses **(B)**. For Hits vs. Alarms, differences were centered also at sensor C2 **(C)** beginning at 324 ms and reaching a maximum *F* value of 66.18 at 549 ms with mean amplitudes being greater for hits than false alarms **(D)**. For Hits vs. Correct Rejections, differences were centered also at sensor C2 **(E)** beginning at 324 ms and reaching a maximum *F* value of 66.18 at 549 ms with mean amplitudes being greater for hits than correct rejections **(F)**.

*Post hoc* pairwise comparisons revealed multiple clusters of significant differences between Hits vs. Misses, Hits vs. False Alarms and Hits vs. Correct Rejections (Table 3). The most significant cluster in each of these three comparisons was centered on sensor C2. For Hits vs. Misses (Table 3, Hits. vs. Misses, Cluster 2, *p* = 0.001) significant differences were centered at sensor C2 (Figure 5A) beginning at 353 ms and reaching a maximum *F* value of 81.33 at 600 ms with mean ERP amplitudes being greater for hits than misses (Figure 5B). For Hits vs. Alarms (Table 3, Hits. vs. False Alarms, Cluster 2, *p* = 0.001) differences were centered also at sensor C2 (Figure 5C) beginning at 324 ms and reaching a maximum *F* value of 66.18 at 549 ms with mean amplitudes being greater for hits than false alarms (Figure 5D). For Hits vs. Correct Rejections (Table 3, Hits. vs. Correct Rejections, Cluster 2, *p* = 0.001) differences were centered also at sensor C2 (Figure 5E) beginning at 324 ms and reaching a maximum *F* value of 66.18 at 549 ms with mean amplitudes being greater for hits than correct rejections (Figure 5F). *Post hoc* pairwise comparisons revealed no significant differences between Misses and False Alarms.

#### Frontal and parietal changes distinguish short- from long-term scenes

**FIGURE 6:**
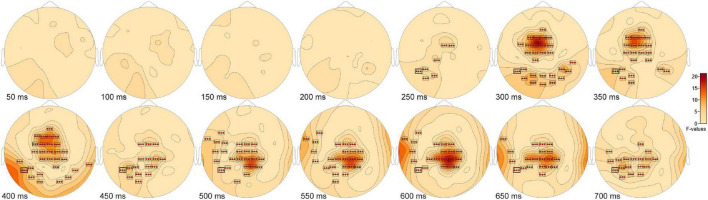
ANOVA F-map for the old scene retention interval comparison. F-map for the comparison of old scenes presented within the three different retention intervals revealed one spatially extended cluster of significant differences (*p* < 0.00001, boxes with three red stars) starting at 228 ms after stimulus onset and reaching a maximum F at 317 ms at sensor FC1.

The ANOVA F-map for the comparison of old scenes presented within the three different retention intervals revealed one spatially extended cluster of significant differences (Table 2, Old_1_ vs. Old_2_ vs. Old_3_, Cluster 1, *p* < 0.00001) and one cluster of marginally significant differences (Table 2, Old_1_ vs. Old_2_ vs. Old_3_, Cluster 1, *p* = 0.045). The significant cluster (*p* < 0.00001, red stars, Figure 6) exhibited differences starting at 228 ms after stimulus onset and reached a maximum F of 20.01 at 317 ms at sensor FC1.

**FIGURE 7:**
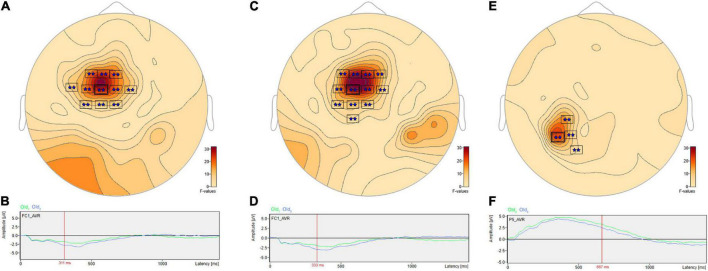
ANOVA *post hoc* pairwise comparisons with higher ERP amplitudes for shorter- vs. longer-term old scene retention intervals. The most significant cluster in each of the three pairwise comparisons, Old_1_ vs. Old_2_; Old_1_ vs. Old_3_; and Old_2_ vs. Old_3_, revealed higher amplitude ERPs for the shorter- vs. longer-term intervals. This includes at sensor FC1 **(A)** a less negative amplitude for Old_1_ compared to Old_2_ beginning at 230 ms and reaching a maximum *F* value of 31.92, *p* = 0.001 at 311 ms **(B)**. It also includes at sensor FC1 **(C)** a less negative amplitude for Old_1_ compared to Old_3_ beginning at 229 ms and reaching a maximum *F* value of 34.76, *p* = 0.001 at 333 ms **(D)**. Finally, it also includes at sensor P5 **(E)** a more positive amplitude for Old_2_ compared to Old_3_ beginning at 635 ms and reaching a maximum *F* value of 22.49, *p* = 0.001 at 667 ms **(F)**.

*Post hoc* pairwise comparisons revealed multiple clusters of significant differences among the Old_1_, Old_2_, and Old_3_ scenes. The most significant cluster in each of the three pairwise comparisons (Table 3, Old_1_ vs. Old_2_; Old_1_ vs. Old_3_; Old_2_ vs. Old_3_) revealed higher amplitude ERPs for the shorter- vs. longer-term intervals (Figure 7). This includes at sensor FC1 (Figure 7A) a less negative amplitude for Old_1_ compared to Old_2_ beginning at 230 ms and reaching a maximum *F* value of 31.92 at 311 ms (Figure 7B). It also includes at sensor FC1 (Figure 7C) a less negative amplitude for Old_1_ compared to Old_3_ beginning at 229 ms and reaching a maximum *F* value of 34.76 at 333 ms (Figure 7D). Finally, it also includes at sensor P5 (Figure 7E) a more positive amplitude for Old_2_ compared to Old_3_ beginning at 635 ms and reaching a maximum *F* value of 22.49 at 667 ms (Figure 7F).

**FIGURE 8:**
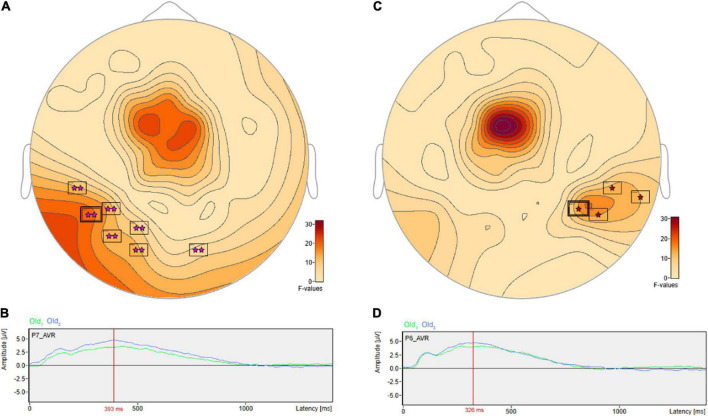
ANOVA *post hoc* pairwise comparisons with higher ERP amplitudes for longer- vs. shorter-term old scene retention intervals. Significant differences wherein the ERP amplitudes were higher for the longer- vs. shorter-term intervals included sensor P7 **(A)** with a more positive amplitude for Old_2_ compared to Old_1_ beginning at 228 ms and reaching a maximum *F* value of 18.9, *p* = 0.001 at 393 ms **(B)**. It also included sensor P6 **(C)** with a more positive amplitude for Old_3_ compared to Old_1_ beginning at 283 ms and reaching a maximum *F* value of 16.93, *p* = 0.024 at 326 ms **(D)**.

There were pairwise comparisons that revealed the opposite pattern: significant differences wherein the ERP amplitudes were higher for longer- vs. shorter-term intervals. This includes at sensor P7 (Figure 8A) with a more positive amplitude for Old_2_ compared to Old_1_ beginning at 228 ms and reaching a maximum *F* value of 18.9, *p* = 0.001 at 393 ms (Figure 8B). It also includes at sensor P6 (Figure 8C) a more positive amplitude for Old_3_ compared to Old_1_ beginning at 283 ms and reaching a maximum *F* value of 16.93, *p* = 0.024 at 326 ms (Figure 8D).

## Discussion

The remarkable human capacity for detailed scene recognition memory has been extensively documented in previous behavioral studies, yet the neural bases supporting this ability remain to be fully understood. Previous neural studies have focused mostly on understanding the basis for scene specificity and therefore have utilized designs in which categorization is the required cognitive task for making decisions about stimulus sets consisting of scenes and other complex visual stimuli like faces, animals, or objects or scenes with faces, animals or objects (Thorpe et al., 1996; Tsivilis et al., 2001; Rousselet et al., 2002; Rousselet et al., 2004; Harel et al., 2016). Understanding categorization ability, although certainly an interesting and highly developed cognitive function, was not the focus of the present study. Instead, the questions addressed here involved scene memory over short (i.e., seconds) and long (i.e., minutes) retention intervals. The first objective was to understand when and how neural patterns distinguish novel, familiar, and scrambled scenes. We therefore included as a baseline condition a set of phase-scrambled scenes in which color and spatial frequency were similar to the real outdoor color scenes. Subjects could not, however, infer from the phase-scrambled scenes anything about place, spatial layout, or meaning from the content of the images. The use of scenes and phase-scrambled counterparts, rather than complex stimuli of different categories, makes the proactive interference experienced during viewing of the interspersed scrambled scenes perceptual rather than categorical.

The first novel contribution of the present study is the characterization of the spatiotemporal neural patterns associated with distinguishing new and old scenes from the phase-scrambled versions. A group ANOVA and *post hoc* paired comparisons revealed occipital and parietal ERPs discriminated new from scrambled and old from scrambled scenes. Inspection of ERP amplitudes showed that this involved greater positivities for new and old scenes compared to the scrambled scenes. Parietal positivities were significantly greater for old relative to scrambled scenes by as early as 270 ms (Table 2) and occipital positivities were greater for new relative to scrambled scenes by 139 ms (Table 2). The greater parietal positivity at 270 ms is slightly longer than a recent finding showing that a P2 amplitude peaking at 220 ms is sensitive to distinguishing open and closed natural scenes (Harel et al., 2016).

The second novel contribution of the present study is the characterization of the spatiotemporal changes associated with discriminating old from new scenes. Paired comparison of New vs. Old ERPs found a larger negativity for New compared to Old starting at 177 ms and reaching maximal difference by 352 ms. In other words, Old ERPs were less negative (i.e., more positive) than New responses at a frontal site, seemingly consistent with the time course for the well-documented FN400 old/new effect, which has been hypothesized to be related to stimulus familiarity (Curran and Cleary, 2003; Curran and Friedman, 2004). We then examined finer distinctions among new and old stimuli using a signal detection breakdown of responses and found strong evidence for a frontocentral positivity whereby hits (old scenes correctly identified as old) evoked stronger ERPs than misses (old incorrectly identified as new), false alarms (new incorrectly identified as old), and correct rejections (new correctly identified as new). This finding is consistent with a separate study of young adults who incidentally encoded and recognized photos of outdoor scenes and found a frontocentral subsequent memory effect with high-confidence hits exhibiting greater positivity compared to misses (Gutchess et al., 2007).

The third novel contribution of the present study concerns the characterization of differences in evoked responses to old scenes as a function of retention interval. The study was designed so that some scene presentations were repeated a second time within a 20 s window after the first presentation. We labeled this second presentation as occurring within a short-term interval since 20 s is often assumed as the temporal limit for short-term memory based on classic interference paradigms (Peterson and Peterson, 1959; Keppel and Underwood, 1962). Although it should be noted that while this assumption is based on paradigms that assess memory based on verbalizable items like letter trigrams, it has been demonstrated using time-frequency that the right parietal region is active during the maintenance (6 s delay) of two scenes in short-term memory (Ellmore et al., 2017). Two other intervals of between 30 s and 3 min, and between 4 and 10 min were classified as longer-term intervals. The behavioral results support a distinction among these three intervals with accuracy highest for short-term recognition and falling significantly for the later intervals but remaining well-above chance.

The ERPs obtained in the present study also support a distinction between short- and longer-term scene memory. Pairwise comparisons revealed higher amplitude ERPs for the shorter- compared to longer-term intervals including, at sensor FC1, a less negative amplitude for Old_1_ (seen within the 20 s interval) compared to Old_2_ (seen within the 30 s and 3 min interval) beginning at 230 ms and reaching maximum at 311 ms. It also included at sensor FC1 a less negative amplitude for Old_1_ compared to Old_3_ (seen within the 4- and 10-min interval) beginning at 229 ms and reaching a maximum at 333 ms. Finally, it included at parietal sensor P5 a more positive amplitude for Old_2_ compared to Old_3_ beginning at 635 ms and reaching a maximum at 667 ms. Thus, the old scene amplitudes that were higher for shorter compared to longer-term retention intervals included two frontal negativities and one parietal positivity. There were significant differences in two parietal sensors in which ERP amplitudes were higher for the longer- compared to shorter-term retention intervals. This included sensor P7 with a more positive amplitude for Old_2_ compared to Old_1_ beginning at 228 ms and reaching a maximum at 393 ms, and also parietal sensor P6 a more positive amplitude for Old_3_ compared to Old_1_ beginning at 283 ms and reaching a maximum at 326 ms. These parietal positivities ended at 517 and 416 ms, respectively, placing them close to the window of the 500–800 ms parietal old/new effect, hypothesized to be related to recollection found with other recognition paradigms (Sanquist et al., 1980; Warren, 1980; Wagner et al., 2005; Rugg and Curran, 2007). These parietal ERP effects for the longer- vs. shorter-term intervals are potentially consistent with recent work showing a rapid and independent role for parietal cortex in a wider network for developing longer-term memory (Brodt et al., 2016).

Dual process models presume that the FN400 frontal negativity and late posterior parietal positivity support familiarity and recognition, respectively, although this hypothesis remains subject to considerable debate. We have found frontal negativities and a parietal positivity at the short- vs. long-term interval comparisons, and two parietal positivities in the longer vs. short-term interval comparisons. A strict interpretation along these lines would be that, at least in this paradigm, short-term scene recognition involves both familiarity and recollection, while longer-term recognition is supported more by recollective like processes. In the present study, we employed a rapid jittered design with stimulus presentation rate of about 6.6 stimuli every 20 s and, because of the speeded presentation, did not attempt to have subjects rate familiarity strength or confidence using a remember/know procedure after each scene presentation. This means that we cannot determine whether the parietal responses evoked by old scenes were enhanced for scenes actually remembered compared to scenes merely recognized as familiar (Warren, 1980). Nevertheless, the results presented here help to establish the rapid intermixed presentation of naturalistic stimuli as a promising paradigm to study the neural basis of the impressive capability of humans for recognizing complex scenes.

In conclusion, we present converging evidence from multiple modalities and analysis approaches that the high-capacity human scene recognition memory system is supported by neural activity patterns occurring as early as 150 ms in widespread occipital, frontal, and parietal regions. Changes occurring later, between 300 and 500 ms, allow a distinction between scenes first presented 20 s ago – the classical putative duration of working memory based on interference studies (Brown, 1958; Peterson and Peterson, 1959) – compared to several minutes ago. These findings provide a baseline by which to evaluate in future neural studies the more nuanced aspects of the scene memory system, including how scene information is consolidated rapidly and available for accurate recognition after even longer retention intervals, including days and beyond (Chandler, 1991), and how neural patterns resist accumulating proactive interference (Makovski and Jiang, 2008) as hundreds or thousands more scenes are encoded for subsequent recognition.

## Data availability statement

The raw data supporting the conclusions of this article will be made available by the authors, without undue reservation.

## Ethics statement

The studies involving human participants were reviewed and approved by the CUNY HRPP. The patients/participants provided their written informed consent to participate in this study.

## Author contributions

TE: conceptualization, funding acquisition, project administration, resources, supervision, visualization, formal analysis, and writing – original draft, review and editing. CR, KN, and NM: data curation, investigation, formal analysis, visualization, and writing – original draft, review and editing. All authors contributed to the article and approved the submitted version.

## Footnotes

1
https://jasp-stats.org/
