# Supplementary material for: Visual continuous recognition reveals behavioral and neural differences for short- and long-term scene memory
Source: Front Behav Neurosci. 2022 Sep 15;16:958609. doi: 10.3389/fnbeh.2022.958609 (PMC9520405; doi:10.3389/fnbeh.2022.958609)
Supplement: Supplementary file 1 [file Data_Sheet_1.pdf]

Supplemental Material

Materials and Methods

Stimuli

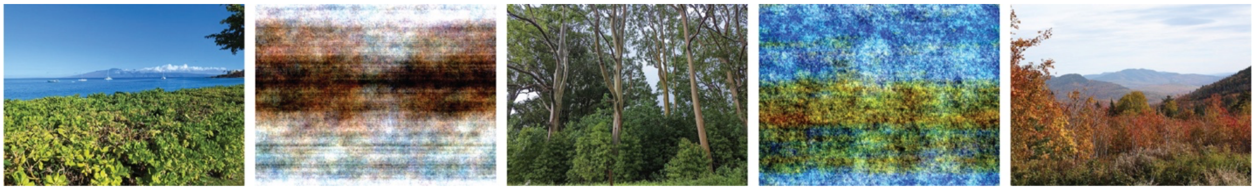

Supplemental Figure 1. Example Stimuli. Three example scenes (not from the SUN database) are shown along with two example phase-scrambled scenes.

Experimental Design

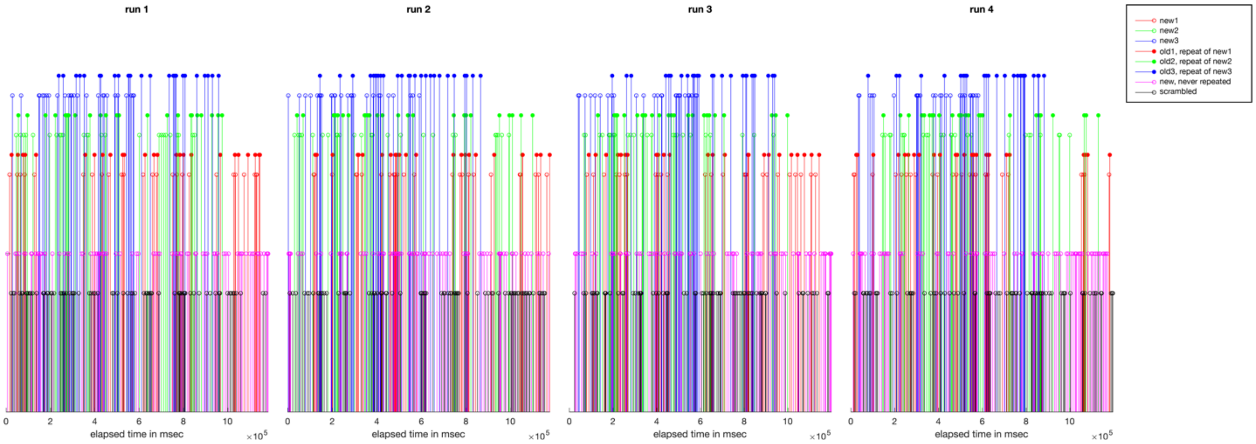

Supplemental Figure 2. Distribution of Stimulus Types Across the Four Runs. Each stimulus type is indicated by a different colored stem plot for each of the four visual continuous recognition task runs that each participant completed during EEG acquisition. Each stem plot shows 20 minutes of the visual continuous recognition task with each stem representing a single stimulus presentation. The height of the stems is varied simply to aide visualization of the different conditions. Three sets of new scenes to be repeated with old_1 occurring in the range of short-term memory (within 20 sec) and old_2 and old_3 occurring in the longer-term range (>30 secs after initial presentation). Also displayed are stems representing phase-scrambled scenes and new scenes only presented once during the 20-minute block of visual continuous recognition task trials.

Results

**Permutation Test Results: All Significant Pairwise Comparisons**											
New vs. Old vs. Scrambled	**Cluster Value**	**p-value**	**Start (ms)**	**End (ms)**	**Max F-Value**	**Latency at Max (ms)**	**Channel at Max**	**Mean New**	**Mean Old**	**Mean Scrambled**	
New vs. Old											
Cluster 1	102951	p=0.00000	1090	1399	38.33	1324	P08	-0.029	-0.037	NA	
Cluster 2	90443	p=0.001	177	876	32.08	352	Fz	-1.717	-0.982	NA	
Cluster 3	38812	p=0.001	194	856	23.03	651	TP10	2.873	2.069	NA	
New vs. Scrambled											
Cluster 1	1082230	p=0.00000	139	1399	121.11	1369	O1	0.074	NA	0.062	
Old vs. Scrambled											
Cluster 1	779757	p=0.00000	270	1399	160.28	684	P3	NA	0.141	0.104	
Signal Detection: Hits vs. Misses vs. False Alarms vs. Correct Rejections											
	**Cluster Value**	**p-value**	**Start (ms)**	**End (ms)**	**Max F-Value**	**Latency at Max (ms)**	**Channel at Max**	**Mean Hits**	**Mean Misses**	**Mean False Alarms**	**Mean Cor. Rej.**
Hits vs. Misses											
Cluster 1	191970	p=0.00000	878	1399	83.99	1287	TP10	-0.078	0.089	NA	NA
Cluster 2	55996	p=0.001	353	749	81.33	600	C2	0.346	-0.398	NA	NA
Cluster 3	8335	p=0.004	568	748	33.98	642	TP10	-1.352	-0.385	NA	NA
Cluster 4	4654	p=0.006	486	669	25.47	541	FT10	-1.784	-0.460	NA	NA
Hits vs. False Alarms											
Cluster 1	225391	p=0.00000	894	1399	66.87	1287	TP10	0.029	NA	0.200	NA
Cluster 2	53681	p=0.001	324	760	66.18	549	C2	0.060	NA	-0.689	NA
Cluster 3	15226	p=0.002	450	736	35.35	650	TP10	-0.143	NA	0.833	NA
Cluster 4	10289	p=0.004	314	426	38.10	363	P8	2.161	NA	3.030	NA
Cluster 5	7027	p=0.006	1034	1378	34.03	1324	FT9	0.871	NA	-0.783	NA
Hits vs. Cor. Rej.											
Cluster 1	110698	p=0.00000	973	1399	49.07	1244	TP10	0.061	NA	NA	0.067
Cluster 2	37555	p=0.001	323	679	44.67	549	C2	-0.090	NA	NA	-0.811
Cluster 3	9725	p=0.001	455	742	33.35	541	FT10	-1.418	NA	NA	-0.257
Cluster 4	2666	p=0.003	321	428	16.18	365	P8	2.682	NA	NA	3.430
Cluster 5	2608	p=0.003	681	745	14.93	710	CPz	0.944	NA	NA	0.435
Cluster 6	558	p=0.005	376	409	12.91	393	PO7	2.462	NA	NA	3.135
Cluster 7	367	p=0.008	548	585	11.04	562	FT9	-1.354	NA	NA	-0.165
Misses vs. Cor. Rej.											
Cluster 1	318	p=0.023	817	845	10.66	842	C5	NA	-0.157	NA	0.492
Cluster 2	303	p=0.024	875	888	13.85	883	C5	NA	-0.383	NA	0.480
False Alarms vs. Cor. Rej.											
Cluster 1	5160	p=0.00000	1230	1376	25.95	1304	C6	NA	NA	1.003	0.394
Cluster 2	2609	p=0.016	1244	1369	24.25	1323	FT9	NA	NA	-0.841	0.465
Short- vs. Longer Retention Intervals: Old$_1$ vs. Old$_2$ vs. Old$_3$											
	**Cluster Value**	**p-value**	**Start (ms)**	**End (ms)**	**Max F-Value**	**Latency at Max (ms)**	**Channel at Max**	**Mean Old$_1$**	**Mean Old$_2$**	**Mean Old$_3$**	
Old$_1$ vs. Old$_2$											
Cluster 1	56142	p=0.00000	947	1399	28.79	1025	CP3	-0.451	0.225	NA	
Cluster 2	25822	p=0.001	230	544	31.92	311	FC1	-1.713	-2.462	NA	
Cluster 3	17603	p=0.001	977	1382	23.16	1258	PO8	0.991	0.089	NA	
Cluster 4	16416	p=0.001	228	517	18.91	393	P7	3.701	4.561	NA	
Cluster 5	4920	p=0.007	532	688	11.18	607	P2	1.993	1.444	NA	
Old$_1$ vs. Old$_3$											
Cluster 1	263782	p=0.00000	868	1399	71.38	1172	Cz	-0.097	NA	0.016	
Cluster 2	75466	p=0.001	229	762	34.76	333	FC1	0.255	NA	-0.466	
Cluster 3	8025	p=0.007	464	674	18.27	601	T7	-0.954	NA	0.258	
Cluster 4	6033	p=0.01	873	1096	20.56	985	FT9	0.833	NA	-0.161	
Cluster 5	3907	p=0.016	474	738	20.59	646	TP10	-1.933	NA	-0.682	
Cluster 6	3590	p=0.02	502	747	20.52	656	FT10	-5.520	NA	-3.684	
Cluster 7	3343	p=0.024	283	416	16.93	326	P6	2.464	NA	3.205	
Old$_2$ vs. Old$_3$											
Cluster 1	21497	p=0.00000	1067	1399	23.22	1343	O2	NA	-0.851	-1.694	
Cluster 2	14781	p=0.001	635	975	22.49	667	P5	NA	1.399	0.797	
Cluster 3	6808	p=0.003	432	633	12.79	571	P5	NA	2.707	2.129	
Cluster 4	4471	p=0.007	1264	1399	15.76	1299	Cz	NA	-0.101	0.551	
Cluster 5	3824	p=0.009	1149	1239	16.66	1186	C2	NA	0.394	1.008	
Cluster 6	2217	p=0.044	823	926	14.91	910	F6	NA	-0.144	0.682	

Supplemental Table. All Significant Pairwise Comparisons. This table lists all the significant pairwise comparisons found within a window of 0 to 1400 ms after stimulus onset.
